# Contribution of Arginine Catabolic Mobile Element and Copper and Mercury Resistance Element in Methicillin-Resistant *Staphylococcus aureus*: A Vantage Point

**DOI:** 10.1155/2022/9916255

**Published:** 2022-10-29

**Authors:** Parya Shokrollahi, Alka Hasani, Mohammad Aghazadeh, Mohammad Yousef Memar, Akbar Hasani, Maryam Zaree, Mohammad Ahangarzadeh Rezaee, Javid Sadeghi

**Affiliations:** ^1^Infectious and Tropical Diseases Research Center, Tabriz University of Medical Sciences, Tabriz, Iran; ^2^Department of Bacteriology and Virology, Faculty of Medicine, Tabriz University of Medical Sciences, Tabriz, Iran; ^3^Clinical Research Development Unit, Sina Educational Research and Treatment Centre, Faculty of Medicine, Tabriz University of Medical Sciences, Tabriz, Iran; ^4^Department of Clinical Biochemistry and Laboratory Sciences, Faculty of Medicine, University of Medical Sciences, Tabriz, Iran

## Abstract

Different clones of community-acquired methicillin-resistant *Staphylococcus aureus* (CA-MRSA) are dominating geographically. One of the significant, hypervirulent, CA-MRSA and a significant health concern clones is USA3000, found worldwide regionally with varying frequencies. The clone harbors several mobile genetic elements (MGEs) including, arginine catabolic mobile element (ACME) and copper and mercury resistance genes (COMER), accomplished by horizontal gene transfer from *S. epidermidis*. Evidence suggests that ACME and COMER have a more prominent role in enhancing biofilm capacity and ultimately persistent infections. This review highlights the comprehensive view on ACME and COMER structure, their distribution, and the mechanism of action along with pathogenetic features of USA3000 encompassing their role in biofilm formation, adhesion, quorum sensing, resistance to antibiotics, chemotaxis, and nutrient uptake. We also provided an insight into the role of ACME and COMER genes in the survival of bacterium. Our results shed light on the emergence of two independent clones possessing ACME (North American) and COMER (South American) elements which later disseminated to other regions. ACME and COMER both are adjacent to staphylococcal cassette chromosome mec type IV (SCCmec IV). The acquisition of mecA, followed by COMER or ACME has been shown as a significant factor in the rise and fall of MRSA strains and their complex ability to adapt to hostile environments. The presence of ACME increases fitness, thereby allowing bacteria to colonize the skin and mucous membrane while COMER contributes to genetic stability by knocking over the copper-mediated killing in macrophages. Evidence suggests that ACME and COMER have a more prominent role in enhancing biofilm capacity and ultimately persistent infections. Interestingly, ACME strains have been shown to possess the ability to counteract skin acidity, thereby allowing increased skin colonization. A profound understanding of MGEs in *S. aureus* plays an important role in the prevention of epidemic clones.

## 1. Introduction

The widespread occurrence and dissemination of *Staphylococcus aureus* (*S. aureus*) have been a topic of concern in scientific and research settings. The organism may populace as a colonizer or be an etiological agent of many infections. The pathogenesis of *S. aureus* depends upon the chromosomal and nonchromosomal virulence factors. *S. aureus* possesses diverse features that account for its role as a successful pathogen triggering various minor and life-threatening infections such as wounds, prostheses, and life-threatening diseases such as sepsis, endocarditis, osteomyelitis, and necrotizing pneumonia at the community and nosocomial level [1, 2]. Some *S. aureus* strains ensue high adaptability to selective pressure on account of their high-level antibiotic resistance and the ability to form biofilm leading to treatment failure, which has forced physicians to face many challenges in therapeutics [3, 4]. Amongst antibiotic-resistant *S. aureus*, methicillin-resistant *S. aureus* (MRSA) is the most clinically important owing to its resistance to all *β*-lactam drugs and on the other hand, causing diverse nosocomial infections with high mortality rates [1, 5, 6]. Depending upon the origin, MRSA can either be community-acquired MRSA (CA-MRSA) or hospital-acquired MRSA (HA-MRSA). ST8-MRSA-IVa (USA300) strain, first identified in the year 2000 in the US as a highly pathogenic and CA-MRSA type [7], has gained fame as a high-level antibiotic-resistant clone possessing the ability to produce Panton–Valentine leucocidin (PVL) and to form adhesive and thick biofilms [1, 7, 8]. USA300, once an endemic USA strain, has expanded to other geographical regions [9, 10], and in terms of geographical dispersion has been reported almost everywhere except Antarctica [10, 11]. USA300 isolates are not confined to only the community level but predominate in a healthcare setting. Originally these isolates were reported resistant only to methicillin, but later resistance to macrolides and fluoroquinolones emerged [10]. Antimicrobial resistance (AMR) is a growing concern among clinically relevant bacteria [12] as they get transferred to other bacteria horizontally by means of mobile genetic elements (MGEs) comprising, plasmids, insertion sequences (ISs), and transposons (Tns). In fact, MGEs are the key elements present as distinct regions of DNA with the ability to move within and/or between bacterial cells and are classified into types based on their properties and their genetic layout [12, 13]. The presence of the arginine catabolic mobile element (ACME) and the copper and mercury resistance (COMER) elements is the inherent epidemiological success of USA300 [14]. Both these genetic elements were associated with two parallel but independent epidemics- ACME in the North American epidemic and a novel COMER in the epidemic which occurred in South America [15].

The arginine catabolic mobile element (ACME) is a 31 kb genomic island, first described as a USA300 North American epidemic (USA300-NAE) clone of MRSA and *S. epidermidis* ATCC12228 [5, 15] ACME harbors the role of enhancing the adaptability of *Staphylococcus* species to colonize the skin and mucous membranes [16]. ACME displays similarity to the staphylococcal cassette chromosome mec (SCCmec) element in a way that it integrates into the staphylococcal chromosome at the attachment site attB, which is flanked by direct repeat sequences and is mobilized by the SCCmec encoded ccrAB genes. Moreover, ACME is known to commonly form a composite island with the SCCmec or SCC-associated genes [5, 17]. The copper and mercury resistance (COMER) mobile element was first described in the USA300 South American epidemic (USA300-SAE) MRSA clone [15]. COMER located in the USA300-SAE chromosome takes over ACME in USA300-NAE adjacent to the SCCmec type IV and contributes to genetic stability, thereby up surging the adaptability and survival of the organism by knocking over the copper-mediated killing in macrophages [15, 18, 19]. This mobile element is also associated with an abortive phage infection system and two main gene clusters, the mer operon composed of the merR/A/B genes and the cop operon composed of the copB/L/mco genes [15, 18]. The emergence of diverse clones of MRSA with mec gene and MGEs like ACME and COMER have led scientists to reconsider the complex nature of this bacteria and its ability to adapt to hostile environments. The earlier spread of MRSA clones harboring ACME and COMER in America and later to other regions requires to halt the wide dissemination. However, preventive measures require a profound understanding of the structure of ACME and COMER, their role in infection, and the mechanism of their action. Thus in this review, we attempted to discuss the role of ACME and COMER in the pathogenesis of *S. aureus*, including its ability to biofilm formation, colonization, and infectivity.

## 2. Distribution of ACME and COMER in *Staphylococci spp*

ACME is widespread among USA300 clone and coagulase-negative staphylococci “CoNS” [20]. ACME is more widely distributed in CoNS, namely, *Staphylococcus haemolyticus*, *Staphylococcus capitis,* and *Staphylococcus epidermidis* [21] but not all species. *Staphylococcus epidermidis*, the most important clinical species of CoNS, is considered a major ACME reservoir. ACME ΔI, a truncated variant of ACME-I, has been the first identified type in *S. epidermidis* and revealed to be prevalent in ST5 methicillin-resistant *S. epidermidis* (MRSE) clinical isolates with SCCmec IVa. The prevalence of ACME-arcA has been reported significantly higher in *S. epidermidis* (45.8%) in comparison to other coagulase-negative *Staphylococcus* (CoNS) species (3.7%) [22]. ACME is a gene locus in USA300, which distinguishes it from other strains of *S. aureus*, and research studies performed suggest that ACME in USA300 is probably derived from *S. epidermidis* through horizontal gene transfer [1, 5, 11, 17, 23, 24]. *S. epidermidis* strains VCU050 and NIHLM049 are considered as potential ACME transmitters to USA300 [17]. ACME is prevalent among methicillin-resistant *Staphylococcus aureus* (MRSA) isolates of sequence type 8 (ST8) and evidence suggests that ACME enhanced the ability of ST8-MRSA-IVa (USA300 clone). The archetypal ACME I-0.1 carried by the USA300 lineage of CA-MRSA does not act as a virulence factor but is associated with enhanced fitness and the ability to colonize the skin and the mucosal surfaces [25, 26]. ACME has been identified in a small number of isolates belonging to other MRSA clones but is widespread among CoNS [20]. While most ST8-MRSA-IVa isolates identified to date contain ACME, it has been identified in only a small number of other MRSA genotypes, including ST5-MRSA-II, ST59-MRSA-IVa, ST97-MRSA-V, ST1-MRSA-IVa, ST5-MRSA-IV, and ST239-MRSA-III. [25, 27–30]. COMER and ACME have not been reported in methicillin-sensitive *S. aureus* (MSSA) [18] except in two ST8-MSSA isolates [20]. ACME has been reported in the ST22-MRSA-IV strain and acquisition is hypothesized that it may have involved horizontal transfer and recombination events between MRSA and CoNS. The presence of ACME may enhance the dissemination of ST22-MRSA-IV, an already successful MRSA clone [20]. Studies have shown that the genes that code ACME (opp-3, arc, speG) have been transferred from *S. epidermidis* to USA300 as a single gene locus [1, 11, 17, 31]. ACME is located adjacent to SCCmec IV in USA300 [9]. The speG gene, which is responsible for tolerating polyamine toxicity, has been added to the ACME site in the final stages of ACME horizontal transfer from *S. epidermidis* to USA300. In Latin America, USA300 (LV-USA300) has become one of the most common infectious clones of MRSA. The LV-USA300 contains a MGE called COMER that encodes copper and mercury resistance and lacks the ACME locus. COMER has not been found in other MRSA clones, including USA300 North America (NA-USA300) [31–33]. In LV-USA300 instead of ACME, COMER is adjacent to SCCmec IV and COMER, similar to ACME, appears to have moved from *S. epidermidis* to LV-USA300 [34].

## 3. Structure and Mechanism of Action of ACME and COMER

### 3.1. Arginine Catabolic Mobile Element (ACME)

ACME is a large genetic locus in *S. aureus* consisting of 33 genes and two operons namely, arc and opp-3. Arc operon (containing arcA, arcB, arcC, arcD, and arcR genes) is involved in arginine metabolism and encodes a complete arginine deaminase pathway (the arc cluster converts L-arginine to CO_2_, ATP, and ammonia). Ammonia production in this pathway provides the ability of bacteria to survive in acidic conditions. The other ACME operon is opp-3 comprised of opp-3A, opp-3B, opp-3C, opp-3D, and opp-3E, which encodes the permease oligopeptide system, and is in fact an ABC transporter in both Gram-positive and Gram-negative bacteria. It plays an important role in adhesion, quorum sensing, resistance to antibiotics, chemotaxis, nutrient uptake, etc. [5, 20, 23].

Genetic sequencing has depicted that *S. aureus* chromosomes comprise an arc cluster and two operons opp (opp -1 and opp-2) that are very different from the arc and opp-3 operons encoded by ACME [5, 20, 23]. The arc cluster in the *S. aureus* chromosome is active only under anaerobic growth conditions, but the ACME-encoded arc cluster is also active under aerobic conditions [35], contributing to an increase in bacterial tolerance in acidic and aerobic conditions. In addition, *S. aureus*, opp-1, and opp-2 operons have been detected in many other bacterial species including both Gram-positive and Gram-negative bacteria and are ABC transporters [20]. They perform important functions such as pheromone transport, peptide nutrient uptake, chemotaxis, and quorum sensing [36]. The ACME locus also contains important genes for polyamine N-acetyl transferase (speG) [9]. The speG gene carried by ACME encodes spermine/spermidine N-acetyl transferase which in return detoxifies polyamines produced by all living organisms as bactericidal substances, and thus, facilitates the bacterium to survive and grow in the presence of polyamines (spermine/spermidine) [17, 37]. Polyamines (spermine/spermidine) are bactericidal compounds obtained from human arginine metabolism in human tissues and help heal wounds and inflammation [6].

In general, polyamines are present in almost all living organisms and the term in fact refers to a group of compounds derived from L-arginine (spermine, spermidine, putrescine, and agmatine) [38, 39]. However, the synthesis pathways of these compounds from L-arginine differ in organisms [40]. Sensitivity to polyamines distinguishes *S. aureus* from other bacteria and even from other living organisms [37].

All *S. aureus* strains are sensitive to polyamines except CA-MRSA USA300 which shows resistance. According to the published literature, the reason for USA300 being resistant to polyamines is the presence of the SpeG gene in ACME. Coding of spermine/spermidine N-acetyl transferase by SpeG results in resistance of this strain to polyamines [17, 37].

So far, five distinct ACME allotypes have been described in staphylococci. Type I harbors the arc and opp-3 operons and type II harbors the arc operon only. ACME types I and II have been described in *S. aureus,* and ACME-I is the most common ACME allotype in USA300 [14, 20, 41]. ACME type III harbors only the opp-3 gene cluster. All three types of ACME (I, II, III) are found in *S. epidermidis* [41, 42]. Two new types of ACME (IV and V) have been identified in oral *S. epidermidis*, that are containing kdp operons (encodes a potassium transporter system). ACME Type IV harbors the arc and the kdp operons, and ACME type V harbors all three arcs, opp-3, and kdp operons. The three characteristic gene clusters; arc, opp-3, and kdp encode an arginine deaminase pathway, an oligopeptide permease ABC transporter, and a potassium ABC transporter, respectively [14, 41].

### 3.2. Copper and Mercury Resistance

COMER has been found in the USA300 Latin American (LV-USA300) clone, which is responsible for encoding copper and mercury-resistant genes [17]. COMER contains two operons mer and cop.

Copper is an important cofactor for catabolic reactions and electron transfer in many bacteria, including *S. aureus*. However, higher levels of copper in the bacterial cell induce hydroxyl radical's (^•^OH) formation, which leads to oxidative damage in the macromolecules and ultimately the death of the bacterial cell. To avoid this, bacterial cells release excess copper through copper transport proteins encoded by cop operon, thereby putting themselves out for a survival condition [23]. In *S. aureus* CsoR (Cu-sensitive operon regulator) is responsible for regulating copper concentration [18] and includes copAZ, copBL, and copBmco operons [43].

All *S. aureus* strains contain the copAZ operon. CopA plays a significant role in the transporting of copper into and out of the cell through membrane proteins, and copZ delivers excess intracellular copper to copper-catalyzing enzymes. However, copBL and copBmco operons are not present in all strains of *S. aureus* and have only been observed in some invasive strains such as MRSA252 and ATCC12600 [23].

**Figure 1:**
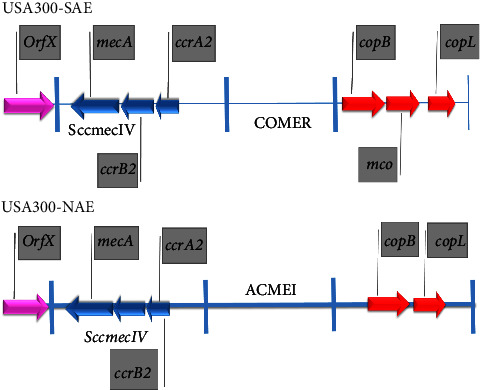
Location of copB (X) and copL genes in NAE-USA300 and SAE-USA300.

The copB mechanisms of action (sometimes known as copX) are like copA and in fact, it is also a copper exporter. CopL prevents the re-entry and adsorption of copper after it is exported by copA and copB. The exact mechanism of mco has not been determined yet [23, 31, 43]. Proper copper homeostasis is essential in pathogenesis because the lack of copAB and copBmco operons in some strains of *S. aureus* leads to the death of the strain by phagocytes and the innate immune system [43]. The copBL operon is the only common operon between ACME (gene locus acquired by NA-USA300) and COMER (GME acquired by LV-USA300) (Figure 1). These two strains have been witnessed epidemically in North and South America in recent years. The joint acquisition of operon copBL by these two strains indicates that high copper tolerance has been implicated in recent North and South American epidemics [18, 43].

Mercury compounds are both organically and inorganically toxic to humans and microorganisms and can bind to membrane proteins and enzymes. Mercury resistance in *S. aureus* is mediated by the mer operon, which contains three genes (merA, merB, merR). The mechanism of inorganic mercury compounds detoxification in *S. aureus* is the reduction of Hg (II+) to Hg (0), which is a reduction reaction in the cytoplasm by the mercury reductase enzyme and finally, Hg0 is released through the outer membrane. The merA gene encodes the enzyme mercury reductase. However, the mechanism of resistance to organic compounds of mercury is mediated by decomposing carbon-mercury bonds with the enzyme mercury lyase. HgII+ is released and then, like the mechanism of inorganic compounds, the HgII+ reduction reaction takes place, and finally, Hg0 in the form of steam leaves the cell. MerB gene encodes mercury lyase to break carbon-mercury bonds in organic mercury compounds. The merR gene regulates the mer operon [23, 44].

## 4. Role of ACME and COMER in Biofilm Formation

Biofilms are aggregates of one or more bacterial species located in an extracellular matrix. Bacterial cells within the biofilm are resistant to killing by antibiotics and the immune system, thus leading to chronic and persistent infections. Therefore, there is great interest in finding methods or strategies to inhibit biofilm formation or treat it.

The biofilm formation by many bacteria occurs generally in four stages comprising adhesion, bacterial cell aggregation, and formation of the microcolony followed by biofilm maturation and dispersal of planktonic cells from the biofilm. Nevertheless, technological advances have shown an additional stage in biofilm formation by *S. aureus* [45]. This stage is called “exodus” which occurs after the multiplication and before the maturation of the biofilm. At the exodus stage, bacterial cells upon reaching confluence, a period of a mass exodus of cells occurs in which a subpopulation of cells is released from the biofilm via Sae-regulated nuclease-mediated eDNA degradation to allow the formation of three-dimensional microcolonies [1, 45].

Biofilm formation in *S. aureus* is a significant strategy for colonization and infection in the body.

### 4.1. Role of ACME in Biofilm Formation

According to different studies, the ability to form biofilms in *S. aureus* strains with ACME locus is higher in comparison to those without ACME [17, 33, 34]. In *S. aureus* USA300, which carries the ACME gene locus, biofilm production has been shown to increase in the presence of polyamines (spermine/spermidine) due to the presence of the speG gene. As mentioned, the speG gene encodes a spermine/spermidine N-acetyltransferase, thereby reducing the toxicity of polyamines, thereby leading to bacterial viability in the presence of polyamines [37]. For this reason, *S. aureus* clones lacking the speG gene are sensitive to polyamines, except for strains such as USA300 that harbor the aforementioned gene [9].

At high concentrations, spermidine increases the transcription of genes involved in biofilm formation. In *S. aureus*, biofilm formation is regulated by an intricate network—the agr system. Polyamine tolerance mediated by speG allows the upregulation of genes required for biofilm formation, increases adherence to abiotic surfaces, and increased resistance to antibiotics and killing by human keratinocytes. Taken together, these data suggest that the acquisition of the speG gene is a crucial factor in the evolutionary success of USA300 [17]. Spermidine also increases the expression of genes encoding fibronectin-binding proteins (fnbA, fnbB), which have been shown to play a role in biofilm formation by *S. aureus* [17, 46]. A study by Machado et al. [47] showed that *S. aureus* strains harboring ACME had more ability to form biofilm compared with other strains without ACME [47]. Regulation of biofilm formation by polyamines such as spermidine has also been observed in Gram-negative bacteria such as *Vibrio cholera* and *Yersinia pestis* [17, 48].

### 4.2. Role of COMER in Biofilm Formation

As mentioned, heavy metals such as copper are toxic in high concentrations to *S. aureus* and can lead to the death of the organism [49]. *S. aureus* strains, which carry COMER remove excess amounts through copper operon due to the presence of copper-resistant genes. And in this way, they have the ability to survive and colonize in the presence of high amounts of copper, which itself can lead to the formation of biofilm. This issue requires more studies.

## 5. Role of MGEs in Host Colonization and Infection

**Figure 2:**
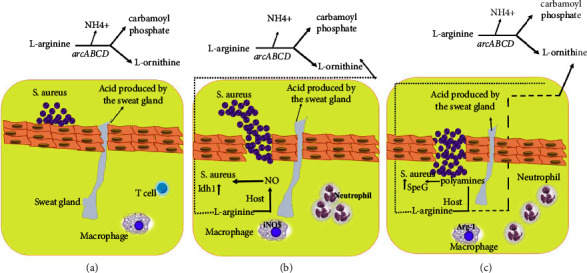
Schematic presentation of the role of ACME in skin survival and colonization by *S. aureus* USA300. (a) *S. aureus* neutralizes the acidic pH of sweat by producing ammonia from L-arginine catabolism and thus colonizes the skin. (b) As the upper layers of the epidermi*s* are broken down by bacteria, the inflammatory phase begins quickly. INOS-producing neutrophils and macrophages are presented at the site of infection. INOS produced by macrophages uses L-arginine to produce NO to kill bacteria and infection. In contrast, *S. aureus* counteracts this innate host attack by reregulating NO-induced lactate dehydrogenase (ldh1). (c) The infection then enters the postinflammatory phase. At this stage, the expression of Arg-1 by host macrophages leads to the synthesis of polyamine production. As mentioned, polyamines are essential for wound healing and inflammation, and most strains of *S. aureus* are sensitive to it, except for strains that have the ACME-encoded speG gene. In *S. aureus*, L-ornithine is produced by the L-arginine arc operon and is transported to the polyamine synthesis pathway, thereby highlighting the role of the speG gene in the organism's survival.

*S. aureus* specifically produces a group of virulence factors for survival and colonization in the host. One of the main physical barriers of skin to prevent bacterial colonization is its acidity (acidic pH). ACME is a virulence factor in some *S. aureus* strains that contain the necessary genes to counteract skin acidity. The arc gene cluster in ACME neutralizes skin acidity by producing ammonia through L-arginine catabolism, so clones with the ACME locus (such as USA300) can tolerate acidic conditions, thereby allowing colonization on the skin. Some pathogens after colonization in the skin can penetrate the underlying layers of the *epidermis* and lead to severe skin and soft tissue infections. In these infections, a proinflammatory phase and a postinflammatory phase occur. In the proinflammatory phase, there are large numbers of phagocytic leukocytes. Also in this phase, the production of iNO$ (derived from L-arginine) by phagocytes increases to which *S. aureus* strains are resistant. In the postinflammatory phase, the production of polyamines from L-arginine increases (Figure 2). Polyamines are normally toxic to *S. aureus* strains, except for USA300 clones that contain ACME-encoded speG. Mutations in speG genes in *S. aureus* have been shown to increase sensitivity to polyamines in the postinflammatory phase of skin and soft tissue infections [9]. Opp-3 is another ACME operon that affects various functions including resistance to antimicrobial peptides, thereby increasing the colonization of bacterial cells on human skin [50]. Evidence suggests that strains carrying the ACME gene locus are more successful in skin colonization and infection than other *S. aureus* strains without ACME [9, 17].

The relationship between the acquisition of some MGEs such as ACME and COMER and the increased abilities of colonization, survival, and infectivity has been reported in *S. aureus* USA300 clone under difficult conditions (e.g., the acidity of the environment and the presence of heavy metals) [18].

## 6. Role of Copper Hypertolerance Genes in Protecting *S. aureus* against Phagocytic Killing

Copper is one of the components of the innate immune system in antibacterial defense [50–52]. Host phagocytes use this micronutrient to kill *S. aureus* intracellularly by actively transferring it into the phagosome. In the eukaryotic cells, the transfer of copper into the cell is facilitated by transport protein CTR1 and its transfer to the phagosome takes place with the help of ATP7a, also known as Menkes protein (MNK), which is a copper-transporting P-type ATPase [19].

In vitro studies have shown that copper deficiency impairs the bactericidal activity of phagocytes, including neutrophils and macrophages [53, 54]. Moreover, copper deficiency in various animals has been shown to expose them to several pathogens including *Salmonella typhimurium*, *Trypanosoma lewisi*, *Candida albicans,* and *Pasteurella hemolytica* [50, 52, 55, 56].

Copper tolerance genes in MRSA strains, including USA300 are carried by MGEs and cause high resistance to copper and bacterial resistance to phagocytic killing [57]. Bacteria are exposed to high levels of copper during infection, which is toxic to them. The mechanism of copper resistance is one of the virulence factors in *S. aureus* to fight the innate immune system. Evidence has shown that mutations in copper-releasing genes in *S. aureus* reduce their virulence [19].

Excess copper under aerobic conditions induces hydroxyl radicals (^•^OH) formation through Fenton and Haber–Weiss reactions, thereby leading to oxidative damage to macromolecules. Copper poisoning under any circumstances leads to the formation of Cu (I)-thiolate bonds, the formation of which leads to damage to important enzymes and proteins such as cluster proteins of iron–sulfur. Host phagocytes e.g., macrophages control the toxic properties of copper [58, 59].

During infection, increased gamma interferon (IFN-*γ*) and plasma copper release stimulate macrophages. Macrophages increase copper imports, resulting in increased bacterial killing by phagocytosis [19]. As discussed, all *S. aureus* strains have a chromosomal copper operon (copAZ) which is responsible for low-level resistance to copper. But copper hypertolerance genes (copBmco), which are present in some strains of *S. aureus*, are carried by various MGEs such as a plasmid, ACME, or COMER. An isogenic mutant of copBmco is not capable of intracellular survival in human macrophages and has little resistance to phagocytic killing in the blood [19].

### 6.1. Mechanism of Copper-Mediated Bacterial Killing by Human Macrophages

**Figure 3:**
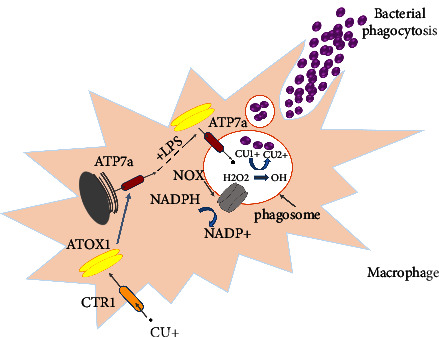
Summary of the mechanism of copper-mediated bacterial killing by human macrophages. (a). Stimulation of CTR1 in the plasma membrane by inflammatory agents such as lipoproteins. (b) Delivery of cytoplasmic copper to the ATP7a copper pump and loading on phagolysosomes by ATOX copper chaperone. (c) Production of superoxide, followed by spontaneous production of hydrogen peroxide by NADPH (NOX) in the phagosome membrane. (d) Increase the bactericidal power of H_2_O_2_ by converting it to hydroxyl radicals via Cu (I)-catalyzed Fenton.

In the first stage, inflammatory factors stimulate the copper transporter 1 (CTR1). CTR1 is located in the plasma membrane and its stimulation leads to the absorption of copper into the cell. Cytoplasmic copper is delivered to the ATP7, a copper pump via the antioxidant protein1 (ATOX1), which loads it into the phagolysosome. NADPH (NOX) in the phagosome membrane produces superoxide, which spontaneously produces hydrogen peroxide (H_2_O_2_). The bactericidal power of H_2_O_2_ increases by converting it to (^•^OH) via Cu (I)-catalyzed Fenton. Cu (I) may also disrupt Fe–S clusters, thereby acting as an antibacterial agent (Figure 3) [59].

Copper hypertolerance genes in *S. aureus* have important implications for society because dominant *S. aureus* clones such as SE-USA300 and LV-USA300 both independently express copper resistance genes in ACME (SE-USA300) and COMER (LV-USA300) [23] which when disseminates may spread the resistance genes and influence their effect.

## 7. Conclusion

*S. aureus* has extraordinary adaptability to survive in the best possible way in diverse environments including human tissues. Acquisition of MGEs such as ACME and COMER in some *S. aureus* strains such as USA300 by horizontal transfer has led to expanded survival, colonization, biofilm production capacity, and prolonged bacterial fitness. In general, the acquisition of ACME (one of the factors involved in skin and soft tissue infections) and COMER (as one of the factors that tolerate harsh environmental conditions such as copper and mercury toxicity) has been a competent genomic advantage for MRSA strains over other bacteria. The genomic transfer of ACME from *S. epidermidis* to MRSA and the positioning of the speG gene proximal to the arc genes have resulted in an increase in the production of the toxic metabolite in human skin. Polyamine tolerance leads to enhanced biofilm formation and adherence, decreased antibiotic susceptibility, and decreased killing by human keratinocytes. The copB locus involved in copper homeostasis/resistance obtained independently as part of the ACME and COMER loci needs further investigation and attention due to the antibacterial properties of copper and its role in innate immunity.

Finally, we propose that the prevalence of ACME and COMER should be attempted to study in MRSA strains obtained from various clinical samples to assess the distribution in hospitals and clinics. A more detailed investigation like whole genome sequencing is required further investigate the possible variants so as to prevent their spread.

## Data Availability

The datasets generated during the current study are available from the corresponding author upon reasonable request.
